# Short-term risk of anaemia following initiation of combination antiretroviral treatment in HIV-infected patients in countries in sub-Saharan Africa, Asia-Pacific, and central and South America

**DOI:** 10.1186/1758-2652-15-5

**Published:** 2012-01-30

**Authors:** Jialun Zhou, Antoine Jaquet, Emmanuel Bissagnene, Beverly Musick, Kara Wools-Kaloustian, Nicola Maxwell, Andrew Boulle, Firas Wehbe, Daniel Masys, Jeniffer Iriondo-Perez, Jay Hemingway-Foday, Matthew Law

**Affiliations:** 1 The Kirby Institute, The University of New South Wales, Sydney, NSW, Australia; 2 INSERM CRE U 897, Institute of Public Health, Epidemiology & Development (ISPED), Université Victor Ségalen Bordeaux, Bordeaux, France; 3 Service de Maladies Infectieuses et Tropicales (SMIT), Centre Hospitalier Universitaire (CHU) de Treichville, Abidjan, Côte d'Ivoire; 4 School of Medicine, Indiana University, Indianapolis, Indiana, USA; 5 School of Public Health and Family Medicine, University of Cape Town, Cape Town, South Africa; 6 School of Medicine, Vanderbilt University, Nashville, Tennessee, USA; 7 RTI International, Research Triangle Park, North Carolina, USA

## Abstract

**Background:**

The objective was to examine the short-term risk and predictors of anaemia following initiation of combination antiretroviral therapy (cART) in HIV-infected patients from the Western Africa, Eastern Africa, Southern Africa, Central Africa, Asian-Pacific, and Caribbean and Central and South America regions of the International Epidemiologic Databases to Evaluate AIDS (IeDEA) collaboration.

**Methods:**

Anaemia was defined as haemoglobin of < 10 g/dL. Patients were included if they started cART with three or more drugs, had prior haemoglobin of > = 10 g/dL, and had one or more follow-up haemoglobin tests. Factors associated with anaemia up to 12 months were examined using Cox proportional hazards models and stratified by IeDEA region.

**Results:**

Between 1998 and 2008, 19,947 patients initiated cART with baseline and follow-up haemoglobin tests (7358, 7289, 2853, 471, 1550 and 426 in the Western Africa, Eastern Africa, Southern Africa, Central Africa, Asian-Pacific, and Caribbean and Central and South America regions, respectively). At initiation, anaemia was found in 45% of Western Africa patients, 29% of Eastern Africa patients, 21% of Southern Africa patients, 36% of Central Africa patients, 15% of patients in Asian-Pacific and 14% of patients in Caribbean and Central and South America. Among patients with haemoglobin of > = 10 g/dL at baseline (13,445), the risks of anaemia were 18.2, 6.6, 9.7, 22.9, 11.8 and 19.5 per 100 person-years in the Western Africa, Eastern Africa, Southern Africa, Central Africa, Asian, and Caribbean and Central and South America regions, respectively. Factors associated with anaemia were female sex, low baseline haemoglobin level, low baseline CD4 count, more advanced disease stage, and initial cART containing zidovudine.

**Conclusions:**

In data from 34 cohorts of HIV-infected patients from sub-Saharan Africa, Central and South America, and Asia, the risk of anaemia within 12 months of initiating cART was moderate. Routine haemoglobin monitoring was recommended in patients at risk of developing anaemia following cART initiation.

## Background

According to World Health Organization (WHO) estimations [1], access to combination antiretroviral treatment (cART) has improved dramatically in low- and middle-income countries with limited resources. At the end of 2009, almost 5.3 million people were receiving antiretroviral therapy in low- and middle-income countries, an increase of more than 1.2 million people from December 2008. In addition, with the newly updated treatment guidelines, the number of people estimated to be in need of cART increased from 10 million to close to 15 million at the end of 2009.

Zidovudine (AZT) was recommended by WHO [2] as a first-line regimen in combination with another nucleoside reverse transcriptase inhibitor (NRTI) and a non-nucleoside reverse transcriptase inhibitor (NNRTI). AZT was also used as an alternate for patients switching from stavudine (d4T) to AZT due to toxicity, or as part of the treatment programme's systematic effort to avoid long-term toxicity issues associated with d4T toxicity [3,4]. The 2010 WHO guidelines recommend that countries using d4T in their first-line regimens phase d4T out and replace it with either AZT or tenofovir in order to prevent long-term toxicity [2].

There have been reports of the short-term tolerability related to the use of AZT, in particular the development or worsening of anaemia. Anaemia was associated with previous clinical AIDS disease or other infection, CD4 count, HIV viral load, female sex, age, and low body mass index [5-7]. Given the recent change in WHO guidelines, there was some concern that rates of anaemia may increase with the transition from d4T-containing regimens to AZT-containing regimens.

The objective was to examine the short-term risk and predictors of anaemia following initiation of cART in HIV-infected patients from the Western Africa (WA), Eastern Africa (EA), Southern Africa (SA), Central Africa (CA), Asian (TA) and Caribbean and Central and South America (CSA) regions of the International Epidemiologic Databases to Evaluate AIDS (IeDEA) collaboration.

## Methods

### Study population: the IeDEA collaboration

The IeDEA initiative of the U.S. National Institutes of Health has established international regional centres for the collection and harmonization of data and the establishment of an international research consortium to address unique and evolving research questions in HIV/AIDS currently unanswerable by single cohorts [8]. Clinically derived HIV treatment data is being collected by researchers throughout the world. This initiative provides a means to establish and implement methodologies to effectively pool the collected data from regions around the globe, thus providing a cost-effective means of generating large data sets to address high-priority research questions related to HIV/AIDS care.

By developing a proactive mechanism for the collection of key variables, this initiative will enhance the quality cost effectiveness and speed of HIV/AIDS research. The sources that support the IeDEA research agenda include independently funded investigators and clinical networks, domestic and international cohorts, individual clinicians caring for large numbers of HIV-infected persons, and national or local databases. Currently, there are seven IeDEA regions: Canada and United States; Caribbean and Central and South America; Asia and Pacific; Western Africa; Central Africa; Eastern Africa; and Southern Africa. Data from more than 300,000 HIV-infected persons from 38 different countries are currently included under this initiative. Details of the IeDEA initiative can be found at http://www.iedea.org/. The IeDEA regions that participated in this analysis were the Western Africa [9], Southern Africa [10] and Eastern Africa [11], Central Africa [12], Asia-Pacific regions [13] and Caribbean and Central and South America [14].

Adult patients (age > 18 years) were included if they initiated cART regimens that contained three or more drugs and had haemoglobin levels above 10 g/dL within 90 days prior to cART initiation, and at least one follow-up haemoglobin test.

### Procedures and statistical analysis

#### Study procedures

The concept for this analysis was reviewed and approved by the IeDEA Executive Committee and all the participating regional steering committees. The data elements in this analysis included baseline and demographic data, HIV disease staging according to

Centers for Disease Control and Prevention (CDC) and/or WHO classification, CD4 and HIV viral load testing, antiretroviral treatment, haemoglobin testing, and weight and height measurements. The regional data centres reviewed and extracted the requested data from their regional databases or requested the identified variables from designated programmes within their regions.

The data were then centrally aggregated and analysed at The Kirby Institute in Sydney, Australia, the regional data centre of the Asia-Pacific IeDEA region. Data consistency checks were conducted when the data were received. This included queries on apparent data-entry errors, out-of-range testing results, antiretroviral treatment combinations that fell outside of the standard of care (for example, AZT concurrent with d4T), and possible data-entry error, such as dates of starting and stopping cART.

The IeDEA Pharmacovigilance and Data Harmonisation Working Groups, comprised of members from each IeDEA region, facilitated the early stage of concept development, as well as later data collection and preparation of the analytical datasets.

#### Statistical analysis

We used the NIH Division of AIDS definitions for Grading the Severity of Adult and Paediatric Adverse Events [15]. Anaemia was defined as a haemoglobin level of < 10 g/dL, and severe anaemia as a haemoglobin level of < 7.5 g/dL.

Mean change of haemoglobin level from cART initiation to 36 months was graphically represented in patients with baseline and follow-up haemoglobin tests. The proportions of patients with anaemia at month 12 after initiation of antiretroviral treatment were tabulated by baseline haemoglobin level and stratified by initial AZT or d4T use. Time to anaemia and severe anaemia within 12 months of cART initiation was assessed by survival analysis. Patients tested but not found to be anaemic were censored at month 12. Factors associated with anaemia were examined using Cox proportional hazards models and stratified by IeDEA region.

Due to the fact that the proportions of patient initiating AZT-containing cART were different across the IeDEA regions, we further investigated the interaction term between AZT use and IeDEA region in predicting anaemia at 12 months. In these analyses, the risk factors for anaemia identified in the main Cox model, and their directions and magnitude, remained largely the same, indicating the robustness of our analyses (data not shown). The analysis was performed using SAS (version 9.1, SAS Institute Inc., Cary, North Carolina, USA) and STATA (version 10.1, StataCorp, College Station, Texas USA).

## Results

The baseline characteristics are shown in Table 1. The number of cohorts contributing patients varied between IeDEA regions: 12 in WA, one in EA, seven in SA, 10 in CA, one in TA, and three in CSA. A total of 19,947 patients initiated cART containing three or more drugs and each had a baseline haemoglobin test and at least one follow-up haemoglobin test (7358 in WA, 7289 in EA, 2853 in SA, 471 in CA, 1550 in TA and 426 in CSA).

**Table 1:** Patient characteristics at cART initiation

	West Africa	Eastern Africa	Southern Africa	Central Africa	Asia-Pacific	Central & South America	Total
No. cohorts in database	12	1	7	10	1	3	34
No. patients in database	14340	8992	3459	18047	4074	1644	50556
No. initiating cART with 3 or more antiretrovirals	12502	8971	3357	4715	3501	1644	34690
No. with haemoglobin at initiation	10823	7326	3265	2215	1754	460	25843
No. with follow up haemoglobin test	7358	7289	2853	471	1550	426	19947
(among patients with haemoglobin at initiation)							
Year cART was initiated							
Median (IQR)	05 (04,06)	05 (05,06)	05 (04,06)	09 (08,09)	04 (02,05)	04 (02,05)	05 (04, 06)
Gender							
Male	2665 (36%)	2876 (39%)	672 (24%)	142 (30%)	1097 (71%)	270 (63%)	7722 (39%)
Female	4693 (64%)	4413 (61%)	2181 (76%)	329 (70%)	453 (29%)	156 (37%)	12225 (61%)
Age (years, at initiation)							
Median (IQR)	37 (31,43)	38 (33,45)	34 (29,40)	39 (33,45)	35 (30,42)	37 (31,44)	37 (31,44)
< = 30	1661 (23%)	1176 (16%)	891 (31%)	72 (18%)	442 (27%)	99 (23%)	4321 (22%)
31~40	3116 (42%)	3054 (43%)	1257 (44%)	166 (36%)	669 (43%)	182 (43%)	8444 (43%)
41+	2581 (35%)	2985 (41%)	705 (25%)	167 (36%)	459 (30%)	145 (34%)	7042 (35%)
Missing	0	74	0	66	0	0	140
Reported exposure							
Heterosexual contact	2127 (100%)	1540 (100%)	2825 (100%)	236 (99%)	1057 (76%)	289 (99%)	8074 (96%)
Homosexual contact	0 (0%)	0 (0%)	0 (0%)	2 (1%)	299 (21%)	0 (0%)	301 (4%)
Injecting drug use	0 (0%)	0 (0%)	0 (0%)	0 (0%)	40 (3%)	3 (1%)	43 (< 1%)
Other/unknown	5231	5749	28	233	154	134	11529
Haemoglobin level at initiation (g/dL, within 90 days before intiation)							
Median (IQR)	10.1 (9.0,11.5)	11.2 (9.6,12.8)	11.2 (10.0,13.0)	10.1 (9.4, 11.8)	12.2 (10.8,13.8)	12.0 (11.0,14.0)	10.9 (9.4,12.4)
> = 10 g/dL	4057 (55%)	5142 (71%)	2257 (79%)	304 (64%)	1317 (85%)	368 (86%)	13445 (68%)
7.5~ < 10 g/dL	2738 (37%)	1773 (24%)	517 (18%)	156 (33%)	206 (13%)	51 (12%)	5411 (27%)
6.5~ < 7.5 g/dL	365 (5%)	224 (3%)	46 (2%)	8 (2%)	18 (1%)	5 (1%)	666 (3%)
< 6.5 g/dL	198 (3%)	150 (2%)	33 (1%)	3 (1%)	9 (1%)	2 (< 1%)	395 (2%)
Haemoglobin test after initiation (up to year one)							
Median number of tests (IQR)	1 (1,2)	1 (1,2)	2 (1,3)	1 (1,2)	3 (1,4)	3 (1,5)	1 (1,2)
Median days from initiation							
to the first test (IQR)	217 (180,337)	212 (163,342)	294 (186,339)	104.5 (14, 210)	282 (194,336)	274 (170,331)	239 (175, 338)
CD4 count at initiation (cells/mm^3^, within 90 days before initiation)							
Median (IQR)	136 (56,220)	101 (44,166)	87 (32,155)	148.5 (62, 228)	112 (35,204)	120 (50,212)	112 (45,187)
< = 50	1662 (24%)	1907 (28%)	904 (34%)	63 (22%)	451 (33%)	92 (27%)	5079 (27%)
51~100	1090 (15%)	1506 (22%)	565 (22%)	38 (13%)	214 (15%)	60 (18%)	3473 (19%)
101+200	2223 (31%)	2512 (36%)	833 (32%)	94 (32%)	263 (26%)	96 (28%)	6121 (33%)
201+	2137 (30%)	946 (14%)	321 (12%)	95 (33%)	364 (26%)	93 (27%)	3956 (21%)
Not available	246	418	230	181	158	85	1318
HIV RNA at initiation (copies/mL, within 90 days before initiation)							
Median	165600	5565	52702	4900	119500	91000	72274.5
(IQR)	31462, 550025	400, 39600	14041, 184563	< 400, 46155	30000, 413273	9000, 160000	17400, 270000
< 400	7 (5%)	5 (50%)	109 (8%)	3 (27%)	31 (5%)	17 (12%)	175 (7%)
400~10,000	10 (8%)	0 (0%)	157 (11%)	3 (27%)	55 (8%)	18 (13%)	240 (10%)
10,001~100,000	34 (26%)	4 (40%)	633 (46%)	3 (27%)	235 (35%)	57 (42%)	966 (41%)
100,001+	81 (61%)	1 (10%)	491 (35%)	2 (19%)	357 (52%)	45 (33%)	977 (42%)
Not available	7226	7279	1463	460	872	289	19947
Disease stage: CDC 3 or WHO 4							
No	3185 (74%)	6048 (90%)	2349 (83%)	160 (34%)	827 (53%)	138 (37%)	12707 (78%)
Yes	1118 (26%)	639 (10%)	491 (17%)	311 (66%)	723 (47%)	236 (63%)	3518 (22%)
Not known	3055	602	13	0	0	52	3722
Tuberculosis co-infection							
No	6369 (87%)	5345 (73%)	2532 (89%)	17 (4%)	1240 (80%)	396 (92%)	15899 (80%)
Yes	989 (13%)	1944 (27%)	321 (11%)	454 (96%)	310 (20%)	30 (7%)	4048 (20%)
Use of TMP-SMX							
No	2949 (40%)	2972 (41%)	---	330 (70%)	741 (48%)	---	6992 (42%)
Yes	4409 (60%)	4317 (59%)	---	141 (30%)	809 (52%)	---	9676 (58%)
Not known	0	0	2853			426	3279
Initial cART combination containing AZT							
No	4787 (65%)	7000 (96%)	2099 (74%)	190 (40%)	938 (60%)	89 (21%)	15103 (76%)
Yes	2571 (35%)	289 (4%)	754 (26%)	281 (60%)	612 (40%)	337 (79%)	4844 (24%)
Initial cART combination containing d4T							
No	2813 (38%)	291 (4%)	760 (27%)	297 (63%)	725 (47%)	341 (80%)	5227 (26%)
Yes	4545 (62%)	6998 (96%)	2093 (73%)	174 (37%)	825 (53%)	85 (20%)	14720 (74%)
Initial treatment combination (top 4 most frequent)							
d4T/3TC/NVP	2414 (33%)	5978 (82%)	415 (15%)	149 (32%)	560 (36%)	46 (11%)	9562 (48%)
d4T/3TC/EFV	1507 (20%)	919 (12%)	1668 (58%)	22 (5%)	111 (7%)	24 (6%)	4251 (21%)
AZT/3TC/EFV	1398 (19%)	54 (< 1%)	441 (15%)	52 (11%)	249 (16%)	261 (61%)	2455 (12%)
AZT/3TC/NVP	392 (5%)	201 (3%)	280 (10%)	224 (48%)	162 (11%)	20 (5%)	1279 (6%)

Most patients included in this analysis started cART in 2004 and 2005, except those from CA, who started more recently (median 2009). There were more female patients in WA, EA, SA and CA compared with TA and CSA, which had a majority of male patients. In each region, more than 40% of patients were aged between 30 and 39 years. The information on exposure was missing in more than 70% of patients from WA and EA; however, heterosexual contact is the most reported category in all regions (Table 1).

At initiation, anaemia and severe anaemia were found in 37% and 8% of patients from WA, 24% and 5% from EA, 18% and 3% from SA, 33% and 3% from CA, 13% and 2% from TA, and 12% and 2% from CSA, respectively (Table 1). Within 12 months of cART initiation, patients from WA, EA and CA had a median of one haemoglobin test, patients from SA had two tests, and patients from TA and CSA had three tests. The median number of days from initiation to the first haemoglobin test was 217, 212, 294, 104.5, 282 and 274 days in patients from WA, EA, SA, CA, TA and CSA, respectively.

At cART initiation, the patients in each region had median CD4 counts between 101 and 148 cells/mm^3 ^and more than half of the patients did not have a baseline HIV RNA test (Table 1). The proportion of patients with either CDC stage three or WHO stage four varies in different regions, from 9% in EA to 66% in CA. Tuberculosis co-infection was reported in all regions, ranging from 7% in CSA to 96% in CA. Use of co-trimoxazole (TMP-SMX) was reported in 60% of patients in WA, 59% in EA and 52% in TA.

**Figure 1:**
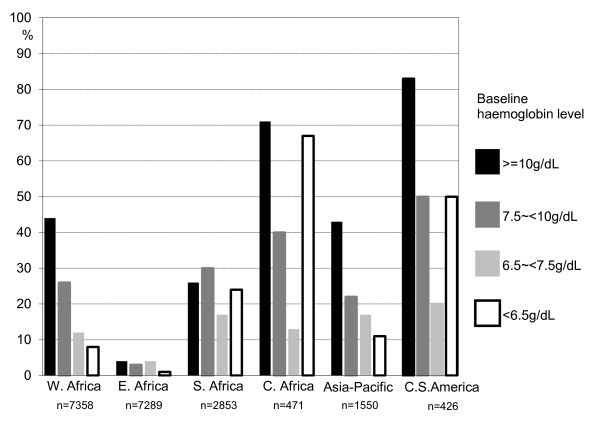
**Proportions of AZT-containing cART by region and baseline haemoglobin level**.

The most frequent cART regimen at treatment initiation was a three-drug combination of two NRTIs (the majority either with d4T+3TC or AZT+3TC), plus one NNRTI (either with NVP or EFV). An AZT-containing regimen was initiated in 35% of patients in WA, 4% in EA, 26% in SA, 60% in CA, 40% in TA, and 79% in CSA. In each of the IeDEA regions, patients with more severe anaemia were generally more likely to initiate with a non-AZT-containing regimen (Figure 1).

**Figure 2:**
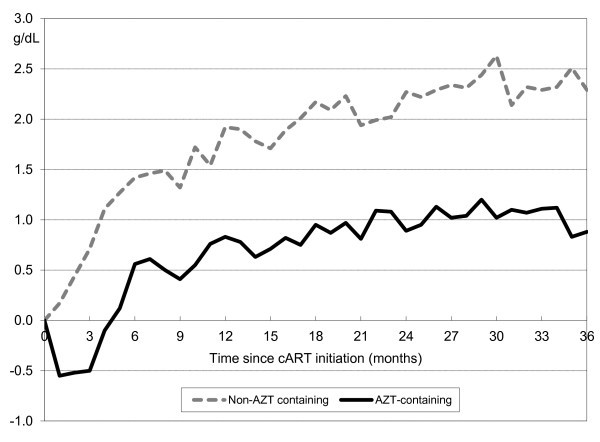
**Mean change of haemoglobin since initiation of cART**.

The mean change in haemoglobin from cART initiation is shown in Figure 2. In patients initiating AZT-containing cART, there was an initial mean haemoglobin decrease of approximately 0.5 g/dL in the first three months; in patients starting with non-AZT-containing cART, there was an immediate haemoglobin increase after initiation. From three months after treatment initiation, there was a mean difference of approximately 1 g/dL between patients initiating AZT-containing cART and those initiating with non-AZT-containing cART that persisted for up to 36 months.

A total of 13,445 (68%) patients initiated cART with normal haemoglobin, 4057 (55%) in WA, 5142 (71%) in EA, 2257 (79%) in SA, 304 (64%) in CA, 1317 (85%) in TA, and 368 (86%) in CSA. Within 12 months of cART initiation, the risks of severe anaemia were 3.9 per 100 person-years (95% confidence interval, CI, 3.6-4.2) and varied from 2.3 (2.0~2.7) in EA to 10.2 (7.1~14.1) in CA; the overall risks of any anaemia (including severe anaemia) were 11.5 per 100 person-years (11.0, 12.1) and varied from 6.6 (5.9~7.4) in EA to 22.9 (31.6~45.3) in CA (Table 2).

**Table 2:** Risk of anaemia (< 10 g/dL) within 12 months after ART initiation among patients with normal haemoglobin (> = 10 g/dL)

	**No**.	Follow up	**No**.	Rate	Univariate analysis		Multivariate analysis	
	patients	(years)	Anaemia	(/100pys)	HR	p value	HR (95% CI)	p value
Total	13445	11893	1373	11.5				
IeDEA region (analyses were stratified by IeDEA region)								
Western Africa	4057	3361	612	18.2				
Eastern Africa	5142	4825	320	6.6				
Southern Africa	2257	2035	197	9.7				
Central Africa	304	214	49	22.9				
Asia-Pacific	1317	1156	136	11.8				
Central & South America	368	302	59	19.5				
Gender								
Male	6000	5344	471	8.8	reference		reference	
Female	7445	6549	902	13.8	1.65	< 0.001	**1.33 (1.18, 1.50)**	**< 0.001**
Age (years, at initiation)								
< = 30	2668	2342	328	14.0	reference		reference	
31~40	5697	5053	577	11.4	0.84	0.014	0.96 (0.83, 1.10)	0.532
41+	4964	4420	455	10.3	0.78	0.001	0.96 (0.83, 1.11)	0.583
Missing	96	78	13	16.7	1.10	0.739	1.16 (0.64, 2.10)	0.622
Baseline haemoglobin (g/dL)								
13+	3826	3552	136	3.8	reference		reference	
12 to < 13	2726	2485	160	6.4	1.67	< 0.001	**1.51 (1.19, 1.90)**	**0.001**
11 to < 12	3389	2961	398	13.4	3.46	< 0.001	**2.96 (2.42, 3.62)**	**< 0.001**
10 to < 11	3504	2895	679	23.4	5.95	< 0.001	**4.94 (4.06, 6.01)**	**< 0.001**
CD4 count at initiation (cells/mm^3^, within 90 days before initiation)								
101+	7138	6408	598	9.3	reference		reference	
51~100	2224	1986	218	11.0	1.18	0.037	**1.24 (1.06, 1.45)**	**0.007**
< = 50	3187	2753	417	15.1	1.62	< 0.001	**1.65 (1.45, 1.87)**	**< 0.001**
Not available	896	746	140	18.8	1.99	< 0.001	**1.71 (1.41, 2.08)**	**< 0.001**
Disease stage: CDC 3 or WHO 4								
No	8775	7930	761	9.5	reference		reference	
Yes	2263	1882	350	17.9	1.54	< 0.001	**1.30 (1.13, 1.50)**	**< 0.001**
Not known	2407	2081	262	12.6	0.80	0.005	0.86 (0.73, 1.00)	0.057
Tuberculosis co-infection								
No	10958	9721	1098	11.3	reference		reference	
Yes	2487	2172	275	12.7	1.20	0.013	0.95 (0.81, 1.10)	0.496
Initial ARV combination containing AZT								
No	9760	8988	716	8.0	reference		reference	
Yes	3685	2905	657	22.6	12.43	< 0.001	**2.51 (2.22, 2.83)**	**< 0.001**
Initial ARV combination containing d4T								
No	3906	3093	677	21.9	reference		reference	
Yes	9539	8800	696	7.9	0.47	< 0.001	0.82 (0.54, 1.25)	0.356
Use of TMP-SMX								
No/Not known	7330	6481	706	10.9	reference		reference	
Yes	6115	5412	667	12.3	1.14	0.032	1.01 (0.89, 1.14)	0.929

Factors associated with developing anaemia 12 months after cART initiation were (Table 2): female gender (33% increase of risk compared with males); low baseline haemoglobin level (significant increase of risk with decreasing haemoglobin at baseline); low baseline CD4 count (significant increase of risk with decreasing CD4 count at baseline); advanced disease stage (30% increase of risk compared with lesser disease stage); initial AZT-containing cART (150% increase of risk compared with patients initiating non-AZT-containing cART). Tuberculosis co-infection and using of TMP-SMX were statistically significant in univariate analysis, but lost significance after adjustment.

**Figure 3:**
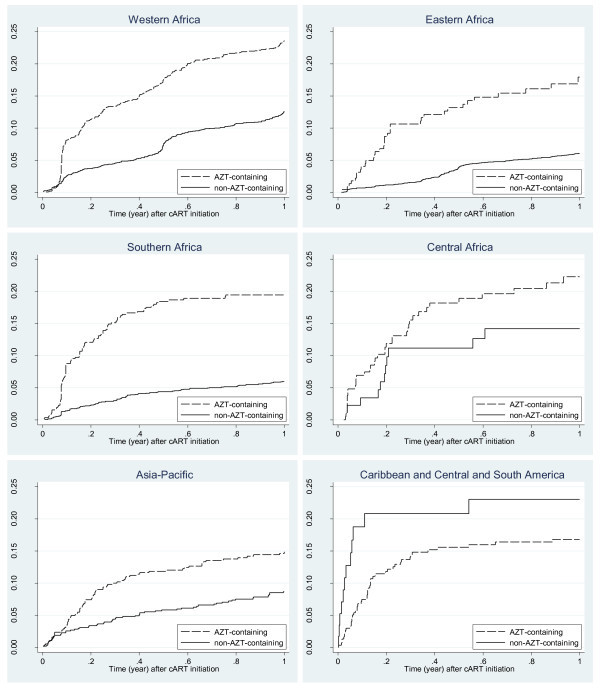
**Risk of anaemia (< 10 g/dL) by initial cART and region**.

Risks of anaemia by initial cART (AZT-containing or not) by IeDEA region are plotted in Figure 3. In patients from WA, EA, SA and TA, patients initiated with an AZT-containing cART had an increased risk of developing anaemia when compared with those initiating a non-AZT-containing regimen (p < 0.001, respectively). The difference between the AZT and non-AZT groups in CA and CSA was not statistically significant after adjusting for the factors just outlined (CA, p = 0.088; CSA, p = 0.396).

Table 3 shows the proportions of patients with anaemia at month 12 after initiation of antiretroviral treatment by baseline haemoglobin level and stratified by initial AZT or d4T use. The table shows that for both AZT and d4T, the proportion of patients with anaemia is associated with baseline haemoglobin level. However, in patients starting with AZT, the proportion of anaemic patients remains high even in patients with baseline haemoglobin from 12 to < 13 g/dL (13%). The proportion of anaemic patients is lower in patients stating d4T in each category of baseline haemoglobin, typically those above 11 g/dL.

**Table 3:** Prevalence of anaemia (< 10 g/dL) at month 12 following ARV initiation

	Initial ARV combination containing AZT		Initial ARV combination containing d4T	
		
	Anaemic at month 12 following ARV initiation			
Baseline haemoglobin (g/dL)	No	Yes	No	Yes
13+	988 (93%)	79 (7%)	2607 (98%)	56 (2%)
12~ < 13	664 (87%)	95 (13%)	1881 (97%)	60 (3%)
11~ < 12	734 (79%)	197 (21%)	2218 (92%)	198 (8%)
10~ < 11	642 (69%)	286 (31%)	2137 (85%)	382 (15%)
< 10	487 (42%)	672 (58%)	3438 (66%)	1743 (34%)

## Discussion

In this study that included six IeDEA regions and 34 HIV treatment cohorts, we found that the risk of developing anaemia within the first year on cART was associated with female sex, low baseline haemoglobin, more advanced immune-deficiency (clinically and immunologically) and receiving an initial cART containing AZT. In addition, we found that d4T-containing regimens were being used more commonly than AZT-containing regimens, especially in patients with severe anaemia at cART initiation.

An initial haemoglobin decrease of approximately 0.5 g/dL in the first three months was observed in patients initiating AZT-containing cART, compared with an immediate haemoglobin increase after initiation in patients starting with non-AZT-containing cART. These data are consistent with results from a meta-analysis of six randomized trials in treatment-naïve patients receiving either AZT or d4T as part of the regimen [16]. In this meta-analysis, haemoglobin levels decreased with AZT-containing treatment by a mean of 0.4 g/dL and 0.2 g/dL at weeks 24 and 48, respectively, but increased with d4T-containing treatment by 0.45 g/dL and 0.58 g/dL, respectively. The DART study also reported low haemoglobin measures at week 4, and grade 4 anaemia (< 6.5 g/dL) occurring at week 12 following initiation of an AZT-containing regimen [17].

The use of AZT+3TC and d4T+3TC as the preferred NRTIs in a regimen has been advocated by WHO since 2000 and are the most common NRTI combinations used in initial HIV treatment regimens in resource-limited settings [18,19]. Until recently, d4T was preferred over AZT due to its lower requirement for laboratory monitoring, lower cost, and availability in fixed-dose combinations tablets, despite its poorer toxicity profile, in association with lactic acidosis, lipodystrophy and peripheral neuropathy. To avoid or minimize the d4T-related long-term toxicity, in 2006, WHO recommended a move away from d4T-containing regimens [20], and in 2009, emphasized this in advice on antiretroviral treatment [21]. This recommendation was in agreement with other treatment guidelines, such as those published by the United States Department of Health and Human Services [22] and the British HIV Association [23]. In settings where d4T-containing regimens were used as the major initial drugs, WHO recommended moves towards AZT- or tenofovir (TDF)-based first-line regimens.

Among patients included in this paper, the median year of cART initiation was 2004-05 in the respective IeDEA regions. The major initial cART regimen contained d4T rather than AZT, which reflects to a large extent the true situation in low- and middle-income countries with limited resources. Moreover, in each of the IeDEA regions, patients with severe anaemia were generally initiated with a non-AZT-containing cART, which was most likely due to the well-established association between anaemia and AZT use [5,16,24].

The crude rate of acquiring anaemia among patients with normal haemoglobin at cART initiation did vary somewhat across the IeDEA regions. This might be due to sampling mechanisms, or biases due to different patterns of haemoglobin testing in each region. For example, at cART initiation and during the course of treatment, patients who had haemoglobin tests might have been selected for testing because they were considered at higher risk of developing anaemia by the local clinician. However, the risks of severe anaemia seemed relatively rare and consistent across the regions. Similar finding was found previously in patients from Uganda and Zimbabwe in the DART trial [17].

Female sex, low baseline haemoglobin, more advanced immune-deficiency (clinically and biologically) and initial cART-containing AZT were associated with the risk of acquiring anaemia within 12 months of starting cART. These risk factors were reported in previous studies [3,5-7,17]. However, the majority of the patients across the IeDEA regions did not have HIV viral load measurement prior to cART initiation, and weight and height data were not generally available across the regions. Consequently, we could not examine the effects of HIV viral load and body mass index on the risk of acquiring anaemia. The use of TMP-SMX was significant in univariate analysis, which might have to do with other infection and the need for prophylaxis since TMP-SMX rarely causes anaemia [25].

We acknowledge several limitations to our study. First, the selection of patients with at least a baseline and one follow-up measurement of haemoglobin might have introduced a bias of selection: patients with documented haemoglobin measurements are more likely to be at risk of anaemia than patients without assessment. In principal, rapid onset of severe, life-threatening anaemia that resulted in loss to follow up and death without a subsequent haemoglobin measurement is a potential scenario in severely resource-limited settings.

Moreover, as this is an observational study, measurements of haemoglobin might not be comparable in every participating country. In addition, important determinants of anaemia, such as body mass index, nutrition intake and malaria status, were not available in the current data assembled for analysis, which made the direct comparison of the risk of anaemia between IeDEA regions difficult, if not impossible, to interpret. Consequently we stratified the region in the Cox regression model to assess the risk factors.

## Conclusions

With the continued rapid scaling up of cART, there is a need to monitor treatment-related toxicity, especially in countries with limited resources and where alternative treatments are not readily available. We found that treating patients earlier, with less immune-deficiency, and with a non-AZT-containing regimen are the only modifiable risk factors associated with anaemia. In countries where TDF-based NRTI regimens are not widely available, a short-term treatment of non-AZT-containing regimens (mostly d4T-containing), followed by a switch to AZT, is worth investigating in terms of efficacy, short- and long-term tolerability, and disease outcomes [26-28]. This could be potentially beneficial for patients at risk of developing anaemia, e.g., female gender, patients with low CD4 counts and patients with advanced disease stage. In addition, routine haemoglobin monitoring is recommended in patients initiating with AZT-containing cART, typically at week 4, 8, 12, or at least every three months [2,17].

## Competing interests

The authors declare that they have no competing interests.

## Authors' contributions

JZ and ML originated the study concept and detailed the analysis plan. JZ performed the data manipulation, statistical analysis, interpretation of results and drafted the manuscript. AJ, EB, BM, KW, NM, AB, FW, DM, JI and JH commented on the study concept and analysis plan, helped interpretation of results and edited the manuscript. All authors have read and approved the final manuscript.
